# Cancer antigen 15-3, platelet distribution width, and fibrinogen in combination to distinguish breast cancer from benign breast disease in non-conclusive mammography patients

**DOI:** 10.18632/oncotarget.18870

**Published:** 2017-06-29

**Authors:** Shuang Fu, Zhi-Yuan Yun, Ming-Ming Cui, Hongxue Meng, Cheng Qian, Tiemin Liu, Zhi-Ping Liu, Rui-Tao Wang, Kai-Jiang Yu

**Affiliations:** ^1^ Department of Internal Medicine, Harbin Medical University Cancer Hospital, Harbin Medical University, Harbin 150081, China; ^2^ Department of Pathology, Harbin Medical University Cancer Hospital, Harbin 150081, China; ^3^ Department of Breast Surgery, Harbin Medical University Cancer Hospital, Harbin 150081, China; ^4^ Division of Hypothalamic Research, Department of Internal Medicine, UT Southwestern Medical Center, Dallas, TX 75390, USA; ^5^ Department of Internal Medicine, University of Texas Southwestern Medical Center, Dallas, TX 75390, USA; ^6^ Department of Intensive Care Unit, Harbin Medical University Cancer Hospital, Harbin Medical University, Harbin 150081, China

**Keywords:** breast cancer, carcinoembryonic antigen, cancer antigen 15-3, platelet distribution width, fibrinogen

## Abstract

Breast cancer is the most frequently diagnosed cancer and the leading cause of cancer death among females. However, mammographic diagnosis is sometimes non-conclusive with a Breast imaging Reporting and Data System (Bi-RaDS) result of 0. Cancer antigen 15-3 (CA15-3) is the most widely used serum tumor marker for breast cancer screening. Platelet distribution width (PDW) is an early indicator of platelet activation. Fibrinogen contributed to angiogenesis and distant metastasis. The aim of this study was to investigate the ability of CA15-3, PDW, and fibrinogen individually or in combination, to distinguish breast cancer from benign breast disease. 200 consecutive patients with breast cancer and 187 patients with benign breast disease were included in this retrospective study. Patients’ characteristics and hematologic tests data at initial diagnosis were collected. The benefit of adding PDW and fibrinogen to a model with only CA15-3 was evaluated as an increased in the area under the curve (AUC) obtained by receiver operating curve (ROC). CA15-3, PDW and fibrinogen are higher in breast cancer patients than in patients with benign breast disease. Single biomarkers had AUC values ranging from 0.687 for fibrinogen to 0.810 for CA15-3. In addition, the combination of PDW, CA15-3, and fibrinogen increased the AUC to 0.900 (0.866-0.928) (p<0.0001), significantly higher than those of any single marker. In conclusion, the combined use of CA15-3, PDW and fibrinogen may be clinically useful in discriminating between breast cancer and benign breast disease in non-conclusive mammography patients.

## INTRODUCTION

Breast cancer is the most frequently diagnosed cancer and the leading cause of cancer death among females worldwide [1]. Although mammography is the gold standard for early breast cancer detection, the overall sensitivity of mammography is reduced in Asian women due to the small volume and high density of breast tissue and mammographic diagnosis is sometimes non-conclusive with a Breast imaging Reporting and Data System (Bi-RaDS) result of 0 [2]. Therefore, identification of novel markers in non-conclusive mammography patients is warranted.

In breast cancer, carcinoembryonic antigen (CEA) and cancer antigen 15-3 (CA15-3) are the two most widely used serum tumor markers in clinical practice. However, these biomarkers that are used for the early detection of breast cancer show limited diagnostic sensitivity and specificity. The American Society of Clinical Oncology (ASCO) guidelines do not currently recommend the use of serum CEA and CA 15-3 for screening, diagnosis, staging, or routine surveillance of breast cancer patients after primary therapy [3, 4].

Activated platelets play a key role in cancer progression and metastases [5, 6]. Mean platelet volume (MPV) is an index of activated platelets and is linked to gastric cancer, ovarian cancer, lung cancer, colon cancer, and breast cancer [7, 8, 9, 10, 11]. Platelet distribution width (PDW), another platelet parameter, indicates variation in platelet size and differentially diagnoses thrombocytopenia [12].

The hypercoagulable state is associated with cancer progression. Fibrinogen, a key coagulation factor, contributed to angiogenesis and distant metastasis. Moreover, increased plasma fibrinogen is associated with poor prognosis in patients with malignancies, such as gastric cancer, lung cancer, colorectal cancer, pancreatic cancer, prostate cancer, renal cell carcinoma, and breast cancer [13, 14]. However, elevated fibrinogen is also observed in inflammation.

Combination of several biomarkers for the early detection may result in enhanced sensitivities and specificities among BI-RADS 0 patients. The aim of the present study was to investigate the ability of CA15-3, PDW, and fibrinogen, individually or in combination, to distinguish breast cancer from benign breast disease in non-conclusive mammography patients.

## RESULTS

The baseline characteristics of the patients are shown in Table 1. The mean age of the patient with benign breast disease and breast cancer was 49.7 (6.2) years and 50.1 (9.5) years, respectively. Postmenopausal women were 125 (66.8%) and 123 (61.5%), respectively. The common pathological type of benign breast disease is breast fibroadenoma (n=106; 56.7%). Most of the patients with breast cancer had low incidence of lymph node metastasis (n = 2; 1.0%) and were diagnosed as T1 (n=168; 84.0%) and stage I (n = 166; 83.0%). There are no patients with distant metastasis in our study. 113 patients (56.5%) with breast cancer were positive ER and positive PR expression. 63 patients (31.5%) with breast cancer were negative ER and negative PR expression. There are 101 patients (50.5%) with breast cancer were negative HER 2 expression and 30 patients (15.0%) with breast cancer were triple-negative patients.

**Table 1:** The characteristics of the participants

	N(%)
Benign breast disease	
Age (years)	
Median (Range)	50 (47–53)
Menopausal status	
Postmenopausal	125 (66.8)
Premenopausal	62 (33.2)
Type	
adenosis	30 (16.1)
Breast fibroadenoma	106 (56.7)
intracanalicular papilloma	38 (20.3)
Others	13 (6.9)
**Breast cancer**	
Age (years)	
Median (Range)	50 (42–56)
Menopausal status	
Postmenopausal	123 (61.5)
Premenopausal	77 (38.5)
Tumor size	
T1	168 (84.0)
T2	30 (15.0)
T3	2 (1.0)
Nodal status	
Positive	2 (1.0)
negative	198 (99.0)
Metastasis	
M0	200 (100.0)
M1	0 (0)
TNM stage	
I	166 (83.0)
II	33 (16.5)
III	1 (0.5)
ER/PR	
+/+	113 (56.5)
+/−	19 (9.5)
−/+	5 (2.5)
−/−	63(31.5)
HER-2	
Negative	101 (50.5)
Positive	99 (49.5)
Triple-negativity	
Triple-negative	30 (15.0)
Non triple-negative	170 (85.0)

ER, estrogen receptor; PR, progesterone receptor; HER-2, human epidermal growth factor-2.

CA15-3, CEA, MPV, PDW and fibrinogen levels in benign breast disease and breast cancer are shown in Table 2. The levels of CA15-3, CEA, PDW and fibrinogen are significantly increased and MPV are markedly reduced in patients with breast cancer compared with the benign cases (p < 0.001).

**Table 2:** The characteristics of the participants between benign group and malignant group

Variables	Malignant group	Benign group	*P* value
Numbers	200	187	
Age (years)	50.1 (9.5)	49.7 (6.2)	0.652
BMI (kg/m^2^)	23.2 (4.2)	23.7 (4.3)	0.273
Menopausal status			
(Postmenopausal, %)	123 (61.5)	125 (66.8)	0.273**
FPG (mmol/L)	5.20 (4.90-5.58)	5.00 (4.70-5.40)	< 0.001*
WBC (×10^9^/L)	6.1 (1.8)	6.4 (1.6)	0.081
Haemoglobin (g/dl)	134.8 (11.9)	132.3 (13.9)	0.057
Platelet (×10^9^/L)	232.3 (49.4)	255.3 (53.2)	< 0.001
MPV (fL)	9.1 (1.1)	9.8 (1.2)	< 0.001
PDW (%)	16.7 (1.0)	14.5 (2.3)	< 0.001
Fibrinogen (g/l)	2.83 (0.66)	2.42 (0.43)	< 0.001
CEA (ng/ml)	1.30 (0.84–1.98)	0.93 (0.48–1.31)	< 0.001*
CA15-3 (U/ml)	9.35 (6.91–12.78)	4.01 (2.61–7.39)	< 0.001*

Values are shown as mean (standard deviation) or median (IQR) or percentage. BMI, body mass index; FPG, fasting plasma glucose; WBC, white blood cell; MPV, mean platelet volume; PDW, platelet distribution width; CEA, carcinoembryonic antigen; CA15-3, cancer antigen 15-3. P-value was obtained using independent samples t-test. *P-value was obtained using Mann-Whitney U test. **P-value was obtained using chi-square test.

We evaluated the levels of CA15-3, CEA, MPV, PDW and fibrinogen levels in the different pathological types of breast masses (Table 3). CA15-3, CEA, MPV, PDW and fibrinogen levels are not markedly different in different types of benign breast disease and breast cancer.

**Table 3:** CA15-3, CEA, MPV, PDW and fibrinogen levels in the different pathological types of breast masses

	N	CA15-3 (U/ml)	CEA (ng/ml)	MPV (fL)	PDW(%)	Fibrinogen (g/l)
**Benign breast disease**						
Adenosis	30	2.50–9.18)	0. 92 (0.62–1.22)	9.7 (0.9)	14.9 (1.9)	2.41 (0.44)
Breast fibroadenoma	106	4.50 (2.60–7.43)	0.94 (0.40–1.32)	9.8 (1.3)	14.6 (2.3)	2.42 (0.46)
Intracanalicular papilloma	38	4.20 (2.58–5.90)	0.84 (0.43–1.36)	9.8 (1.3)	14.2 (2.4)	2.43 (0.39)
Others	13	3.10 (1.70–7.20)	0.95 (0.76–1.37)	10.0 (0.9)	13.7 (2.5)	2.38 (0.31)
*P value*		0.751*	0.840*	0.842	0.334	0.982
**Breast cancer**						
Luminal-A	73	9.30 (6.90–11.80)	1.34 (0.83–1.89)	9.0 (1.1)	16.8 (0.9)	2.82 (0.67)
Luminal-B	64	10.30 (6.65–14.55)	1.26 (0.87–2.05)	9.1 (1.0)	16.5 (1.0)	2.76 (0.66)
HER2(+)	33	9.60 (7.35–12.35)	1.50 (0.95–2.09)	9.2 (0.8)	16.6 (1.0)	2.98 (0.49)
ER(−) PR(−) HER2(−)	30	9.05 (6.60–12.78)	1.10 (0.79–1.81)	9.2 (1.4)	16.9 (1.3)	2.82 (0.78)
*P value*		0.865*	0.691*	0.836	0.276	0.473

CEA, carcinoembryonic antigen; CA15-3, cancer antigen 15-3; MPV, mean platelet volume; PDW, platelet distribution width. P-value was obtained using one-way ANOVA test. *P-value was obtained using KruskaleWallis H test.

Logistic regression analysis was performed to evaluate the risk factors for distinguishing benign breast disease from breast cancer. The risk factors found to be significantly associated with differentiation of benign breast disease and breast cancer in the regression analysis included PDW, fibrinogen, CEA, and CA15-3 (Table 4). In contrast, MPV was found to be inversely associated with differentiation of benign breast disease and breast cancer.

**Table 4:** Multiple logistic regression analysis of factors used for differentiation breast cancer from benign breast disease

Variables	β	OR (95% CI)	*p* value
FPG (mmol/L)	0.285	1.330 (0.868–2.038)	0.19
WBC (×10^9^/L)	−0.149	0.862 (0.706–1.052)	0.144
Haemoglobin (g/dl)	0.014	1.014 (0.989–1.039)	0.277
Platelet (×10^9^/L)	−0.005	0.995 (0.988–1.002)	0.127
MPV (fL)	−0.344	0.709 (0.544–0.925)	0.011
PDW (%)	0.98	2.665 (1.923–3.693)	< 0.001
Fibrinogen (g/l)	1.271	3.563 (1.985–6.395)	< 0.001
CEA (ng/ml)	0.611	1.843 (1.223–2.778)	0.003
CA15-3 (U/ml)	0.174	1.190 (1.115–1.271)	< 0.001

OR, odds ratio; CI, confidence interval. CEA, carcinoembryonic antigen; CA15-3, cancer antigen 15-3; MPV, mean platelet volume; PDW, platelet distribution width.

**Figure 1:**
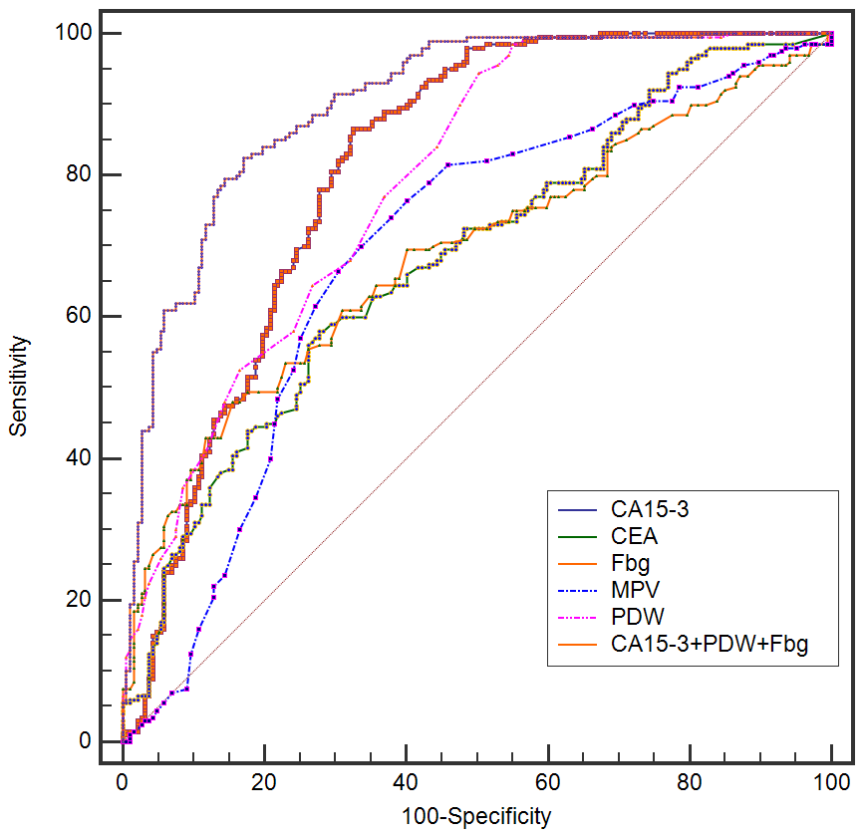
The ROC of CA15-3, CEA, MPV, PDW, and fibrinogen in combination to distinguish breast cancer from benign breast disease in non-conclusive mammography patients

In Table 5, the sensitivity, specificity, positive predictive value, negative predictive value, and area under curve (AUC) values are presented for CA15-3, CEA, MPV, PDW, fibrinogen, the combination of CA15-3, MPV, and fibrinogen, the combination of CA15-3, PDW, and fibrinogen. When used to analyze benign breast masses versus breast cancer, PDW had the highest sensitivity (94.5%), but at the cost of an unsatisfactory low specificity (49.7%). In contrast, fibrinogen had the highest specificity (84.5%) with a low sensitivity (48.0%). The specificity of PDW and the sensitivity of fibrinogen markedly increased when the combination of PDW, CA15-3, and fibrinogen were applied. Single biomarkers had AUC values ranging from 0.680 for CEA to 0.810 for CA15-3; the combination of PDW, CA15-3, and fibrinogen increased the AUC to 0.900 (p < 0.0001) (Figure 1).

**Table 5:** Receiver operating characteristic curve analyses showing the utility of alone or combined markers for differentiating of benign breast disease and breast cancer

Tumor marker	SEN	FNR	SPE	FPR	PPV	NPV	AUC
CEA (ng/ml)	58	42	72.2	27.8	69	61.6	0.680 (0.631-0.727)
CA15-3 (U/ml)	86.5	13.5	67.4	32.6	73.9	82.4	0.810 (0.768-0.848)
MPV (fL)	76.5	23.5	59.9	40.1	67.1	70.4	0.688 (0.640-0.734)
PDW (%)	94.5	5.5	49.7	50.3	66.8	89.4	0.789 (0.744-0.828)
Fbg (g/l)	48	52	84.5	15.5	76.8	60.3	0.687 (0.638-0.733)
CA15-3+MPV+Fbg	82	18	74.3	25.7	77.4	79.4	0.837 (0.796-0.872)
CA15-3+PDW+Fbg	82.5	17.5	82.9	17.1	83.8	81.6	0.900 (0.866-0.928)

SEN, sensitivity; FNR, false negative rate; SPE, specificity; FPR, false positive rate; PPV, positive predictive value; NPV, negative predictive value; AUC, area under curve; CEA, carcinoembryonic antigen; CA15-3, cancer antigen 15-3; MPV, mean platelet volume; PDW, platelet distribution width; Fbg, fibrinogen. The cutoff values of CEA, CA15-3, fibrinogen, MPV and PDW were 1.17 ng/ml, 5.77 U/ml, 2.79 g/l, 9.6 fL, and 15.7 %, respectively.

## DISCUSSION

Our study showed that CA15-3, PDW and fibrinogen are higher in breast cancer patients than in patients with benign breast disease. Moreover, noninvasive blood tests utilizing CA15-3, PDW and fibrinogen, have the potential to significantly enhance the ability to discriminate between breast cancer and benign breast disease in the clinical setting.

Despite best current medical and surgical treatment, the overall prognosis of patients with breast cancer remains poor. Numerous studies point to the key roles of platelet activation in tumor progression. Thrombocytosis is linked with reduced survival in patients with various tumor types, including cancer of the lung, ovary, endometrium, rectum, kidney, stomach, pancreas, brain, and breast. Recent studies revealed that cancer-associated thrombocytosis was a paraneoplastic phenomenon. Tumors could promote platelet production and activation through the interleukin (IL)-6 pathway [15].

In breast cancer, platelet-derived growth factor (PDGF) beta-receptor expression significantly correlates with less favorable clinicopathological parameters and shorter survival [16]. Further study revealed that platelet-derived growth factor-D contributes to aggressiveness of breast cancer cells by up-regulating Notch and NF-κB signaling pathways [17]. Several clinical studies have observed the platelet activation in patients with breast cancer [18]. In line with previous findings, our study indirectly confirmed the results using a simple marker of platelet activation. These data are also consistent to the current knowledge that anti-platelet is considered to be a part of cancer adjuvant therapy [19].

A specific mechanism to explain the role of PDW in breast cancer remains to be determined. Bone marrow cells (including megakaryocytes) dys-regulation may contribute to changed PDW. Platelet distribution width is a measure of platelet heterogeneity. The heterogeneity in platelet volume is caused by heterogeneous demarcation of megakarocytes [20]. Platelet volume is determined both during megakaryopoiesis and during thrombopoiesis. Megakaryocytic maturation, platelet production and platelet size could be modulated by cytokines, such as interleukin-6 (IL-6), granulocytes colony stimulating factor (G-CSF) and macrophage colony stimulating factor (M-CSF) [21]. Increased interleukin-6 (IL-6) has been found in almost all types of tumors acting as a major cytokine in the tumour microenvironment. An increasing body of evidence suggests that IL-6 promotes tumorigenesis by regulating apoptosis, survival, proliferation, angiogenesis, metastasis and metabolism [22]. Furthermore, megakaryopoiesis and subsequent thrombopoiesis in cancer may be stimulated by the cytokines G-CSF and M-CSF, which could be secreted by tumor cells [23]. Another possible mechanism is that platelets play a crucial role in promoting the hypercoagulable state in cancer. Activated platelets create a procoagulant micro-environment that enables the tumor cells to cover themselves with platelets and evade the host immune system. Anandi VL *et al* demonstrated that platelet-activating factor promotes motility in breast cancer cells [24]. In addition, Toth B *et al* confirmed the key role of platelet-derived microparticles in the microenvironment of tumor tissue in breast cancer patients [25]. Those findings are consistent with our results that fibrinogen is elevated in patients with breast cancer.

BI-RADS category 0 is defined as an incomplete assessment and additional examination is necessary. Furthermore, a combination of ultrasonography and mammography is associated with a high rate of false-positive results [2]. However, for the diagnosis of breast cancer, few tumor markers are highly sensitive or specific. Our study revealed that the AUC values for discriminating breast cancer patients from benign breast masses were 0.680 for CEA to 0.810 for CA15-3, respectively. The combination of PDW, CA15-3, and fibrinogen increased the AUC to 0.900, significantly higher than those of any single marker. Moreover, CA15-3, PDW and fibrinogen levels are easily available in the clinical setting. Therefore, a combination of three serum markers is a more comprehensive parameter for breast cancer detection than single index in non-conclusive mammography patients.

In conclusion, the study showed that the combined use of CA15-3, PDW and fibrinogen may be clinically useful in discriminating between breast cancer and benign breast disease. Further studies are needed to validate these results in routine practice.

## MATERIALS AND METHODS

### Study population

The records of 200 consecutive patients with breast cancer and 187 patients with benign breast disease who were admitted to Harbin Medical University Cancer Hospital, Harbin Medical University between Jan 2012 and Dec 2015 were reviewed. Patients meeting all of the following requirements were eligible for enrollment: (1) all patients with breast cancer undergone complete surgical resection and diagnosis was confirmed by histology; diagnosis of benign breast disease was based on breast biopsies; (2) untreated before diagnosis; (3) undergone mammography screening and Bi-RaDS result was 0; (4) measurement of CA15-3 and fibrinogen before surgery. Exclusion criteria included: pregnancy, breast-feeding or non-cancer-related illnesses that precluded surgical tumor resection, hematological disorders, coronary artery disease, hypertension, diabetes mellitus, and medical treatment with anticoagulant, statins, and acetylic salicylic acid. 187 patients with benign breast disease were matched for age, gender, body mass index (BMI), and smoking status.

Written informed consents were obtained from all patients. This study was approved by the Institutional Review Board of Harbin Medical University Cancer Hospital.

### Clinical examination and biochemical measurements

All the subjects underwent physical examination. Body mass index (BMI) was calculated as the ratio of weight (kg) to height squared (m^2^). Clinical data including smoking status, medical history and medication use were recorded for each subject. Venous blood samples after a 10-hour overnight fasting were collected from the individuals within 1 week prior to surgery. The serum carcinoembryonic antigen (CEA) and CA15-3 were measured using an automatic electrochemistry luminescence immunoassay system (ROCHE cobas 8000; Roche, Germany). Fibrinogen is assayed via Clauss method by the automatic coagulometer (Symex CS5100, Japan). Platelet indices were measured by an autoanalyzer (Sysmex XE-2100, Kobe, Japan). The whole blood samples were collected in EDTA-containing tubes, and all samples were processed within 30 minutes after blood collection. The inter- and intra-assays coefficients of variation (CVs) of all these assays were below 5%. The ideal cutoff values for the CEA, CA15-3, fibrinogen, MPV and PDW were determined applying receiver operating curve analysis. The cutoff values of CEA, CA15-3, fibrinogen, MPV and PDW were 1.17 ng/ml, 5.77 U/ml, 2.79 g/l, 9.6 fL, and 15.7 %, respectively.

### Statistical methods

All statistical analyses were performed using SPSS Statistics version 22.0 (SPSS Inc., Chicago, IL, USA). The descriptive statistics are presented as means ± SD or medians (interquartile range) for continuous variables and percentages of the number for categorical variables. When baseline characteristics between two groups were compared, normally distributed continuous variables were compared with the Student t test and skewed-distributed with the Mann-Whitney U test. When baseline characteristics among four groups were compared, normally distributed continuous variables were compared with the one-way ANOVA and skewed-distributed with Kruskal-Wallis H test. The Chi-square test was used for categorical variables. Receiver-operating characteristics (ROC) curve analysis was performed to identify cut-off values of measured serum markers, and the differences in the area under the curve (AUC) were detected by using MedCalc version 15.0. A two-tailed p value less than 0.05 was considered significant in all tests.

## References

[1] Torre LA, Bray F, Siegel RL, Ferlay J, Lortet-Tieulent J, Jemal A (2015). Global cancer statistics, 2012. CA Cancer J Clin.

[2] Yang B, Xu Q, Wu F, Liu F, Ye X, Liu G, Shao Z, Meng X, Mougin B, Wu J (2010). Using peripheral blood mRNA signature to distinguish between breast cancer and benign breast disease in non-conclusive mammography patients. Cancer Biol Ther.

[3] Cardoso F, Saghatchian M, Thompson A, Rutgers E (2008). Inconsistent criteria used in American Society of Clinical Oncology 2007 update of recommendations for the use of tumor markers in breast cancer. J Clin Oncol.

[4] Harris L, Fritsche H, Mennel R, Norton L, Ravdin P, Taube S, Somerfield MR, Hayes DF, Bast RC (2007). American Society of Clinical Oncology 2007 update of recommendations for the use of tumor markers in breast cancer. J Clin Oncol.

[5] Bambace NM, Holmes CE (2011). The platelet contribution to cancer progression. J Thromb Haemost.

[6] Goubran HA, Stakiw J, Radosevic M, Burnouf T (2014). Platelet-cancer interactions. Semin Thromb Hemost.

[7] Kılınçalp S, Ekiz F, Başar O, Ayte MR, Coban S, Yılmaz B, Altınbaş A, Başar N, Aktaş B, Tuna Y, Erbiş H, Uçar E, Erarslan E, Yüksel O (2014). Mean platelet volume could be possible biomarker in early diagnosis and monitoring of gastric cancer. Platelets.

[8] Kemal Y, Demirag G, Ekiz K, Yucel I (2014). Mean platelet volume could be a useful biomarker for monitoring epithelial ovarian cancer. J Obstet Gynaecol.

[9] Kumagai S, Tokuno J, Ueda Y, Marumo S, Shoji T, Nishimura T, Fukui M, Huang CL (2015). Prognostic significance of preoperative mean platelet volume in resected non-small-cell lung cancer. Mol Clin Oncol.

[10] Li JY, Li Y, Jiang Z, Wang RT, Wang XS (2014). Elevated mean platelet volume is associated with presence of colon cancer. Asian Pac J Cancer Prev.

[11] Gu M, Zhai Z, Huang L, Zheng W, Zhou Y, Zhu R, Shen F, Yuan C (2016). Pre-treatment mean platelet volume associates with worse clinicopathologic features and prognosis of patients with invasive breast cancer. Breast Cancer.

[12] Kaito K, Otsubo H, Usui N, Yoshida M, Tanno J, Kurihara E, Matsumoto K, Hirata R, Domitsu K, Kobayashi M (2005). Platelet size deviation width, platelet large cell ratio, and mean platelet volume have sufficient sensitivity and specificity in the diagnosis of immune thrombocytopenia. Br J Haematol.

[13] Kaito K, Otsubo H, Usui N, Yoshida M, Tanno J, Kurihara E, Matsumoto K, Hirata R, Domitsu K, Kobayashi M (2015). Prognostic role of pretreatment plasma fibrinogen in patients with solid tumors: A systematic review and meta-analysis. Cancer Treat Rev.

[14] Yu W, Wang Y, Shen B (2016). An elevated preoperative plasma fibrinogen level is associated with poor overall survival in Chinese gastric cancer patients. Cancer Epidemiol.

[15] Lin RJ, Afshar-Kharghan V, Schafer AI (2014). Paraneoplastic thrombocytosis: the secrets of tumor self-promotion. Blood.

[16] Paulsson J, Sjöblom T, Micke P, Pontén F, Landberg G, Heldin CH, Bergh J, Brennan DJ, Jirström K, Ostman A (2009). Prognostic significance of stromal platelet-derived growth factor beta-receptor expression in human breast cancer. Am J Pathol.

[17] Ahmad A, Wang Z, Kong D, Ali R, Ali S, Banerjee S, Sarkar FH (2011). Platelet-derived growth factor-D contributes to aggressiveness of breast cancer cells by up-regulating Notch and NF-κB signaling pathways. Breast Cancer Res Treat.

[18] Kedzierska M, Czernek U, Szydłowska-Pazera K, Potemski P, Piekarski J, Jeziorski A, Olas B (2013). The changes of blood platelet activation in breast cancer patients before surgery, after surgery, and in various phases of the chemotherapy. Platelets.

[19] Mezouar S, Frère C, Darbousset R, Mege D, Crescence L, Dignat-George F, Panicot-Dubois L, Dubois C (2016). Role of platelets in cancer and cancer-associated thrombosis: experimental and clinical evidences. Thromb Res.

[20] Paulus JM (1981). Recent advances in the story of megakaryocyte physiology. Pathol Biol (Paris).

[21] Kaushansky K (1998). Growth factors and hematopoietic cell fate. A new feature: controversies in hematology. Blood.

[22] Kumari N, Dwarakanath BS, Das A, Bhatt AN (2016). Role of interleukin-6 in cancer progression and therapeutic resistance. Tumour Biol.

[23] Kowanetz M, Wu X, Lee J, Tan M, Hagenbeek T, Qu X, Yu L, Ross J, Korsisaari N, Cao T, Bou-Reslan H, Kallop D, Weimer R (2010). Granulocyte-colony stimulating factor promotes lung metastasis through mobilization of Ly6G+Ly6C+ granulocytes. Proc Natl Acad Sci U S A.

[24] Anandi VL, Ashiq KA, Nitheesh K, Lahiri M (2016). Platelet-activating factor promotes motility in breast cancer cells and disrupts non-transformed breast acinar structures. Oncol Rep.

[25] Toth B, Liebhardt S, Steinig K, Ditsch N, Rank A, Bauerfeind I, Spannagl M, Friese K, Reininger AJ (2008). Platelet-derived microparticles and coagulation activation in breast cancer patients. Thromb Haemost.

